# Selections

**Published:** 1860-07

**Authors:** 


### Selections.

### BUCCAL INFLAMMATIONS.

### *Diseases of Crystal-and-Glass Cutters; Monograph of a Peculiar Buccal Inflammation not hitherto described.*

(Translated for the Dental Register, from "L'Art Dentaire.")

BY DR. PUTEGNAT, OF LOUISVILLE.

M. Putegnat calls the attention of medical men to a disease of the gums, peculiar to certain artisans, that has not before been described. After having inspected the workshops of the *Crystalry* at Baccarat, and having examined the conditions of the male and female operatives in these glass works, M. Putegnat makes the following monograph of an inflammation of the gums, not before known:

"A loathsome affection, little serious in itself, but liable to serious consequences, as we shall hereafter prove, is so common in certain crystal works at Baccarat, that, without any fear of exaggeration, we may affirm that, if all the operatives in these shops do not present traces of it, 95 in 100 of them have it developed to a considerable degree. The man of the best constitution, the most robust, derives no advantage from his temperament; he whose complexion is vermilion; who, in all other respects, enjoys excellent health; who commits no excesses, who is temperate, well fed, healthfully lodged, in easy circumstances, and who neither chews nor smokes; he is no more exempt from this disease of the gums, than the cutter who is lean, bloodless, lymphatic, dissolute, poor, badly fed, miserably lodged, and who makes an excessive use of tobacco. It is enough that they are both cutters at Baccarat. At the end of a sojourn of three months in the cutting shop, they exhibit traces of the malady; and at the end of six months, the symptoms become unmistakable.

"In general, this affection commences and is more severe on the upper jaw. Why this predilection? I do not know. The mucus (mucous membrane?) reddens; its tint becomes a blackish blue, more deep as it approaches the edges bordering on the teeth; and this color forms kinds of arches that represent the alveoles. It is not that which is found on the gums of workers in lead, and it is extremely difficult to give an idea of the difference which exists between these two morbid colorations; actual inspection or the painter's brush alone can do it; so that he who has seen the narrow bluish border of the gum which is the result of poisoning by lead, will not confound it with the arches of a peculiar shade, and of which the interdental pillars are more dark as they approach the free border which this inflammation presents. From what we have just said, it is also manifest that this fascia does not resemble that nacreous one which, according to MM. Ranque, Négrī, and Michel Levy, is a certain sign of the arrival of atoxy in pneumonia.

"The gums swell, especially toward their dental border, which forms a festooned pad. They but rarely furnish tartar, and even then in small quantity; but an acid secretion which, escaping from their free border, is not long in impairing the enamel of the teeth. From thence it results that the anterior or external portion of the teeth, especially the incisive and the superior, where they join the gum, appear at first uneven, then punctured with blackish points, and at last of a dirty black. This color indicates the necrosis of the osseous part of the tooth, and consequently the destruction of the enamel. Arrived at this stage of alteration, the tooth wears away at the neck, particularly from without inward by caries, and ends by breaking off level with the alveolus.

"Thus, in the case of these artisans, the teeth, unlike what happens in scurvy and certain inflammations of the gums, in consequence of the shrinking of the gum and the impairment of the interalveolar mucus (mucous membrane?) do not become bared, nor get loose and fall out, but break off even with the socket. It is thus evident why these men, after a few years' stay in the cutting shops, have, instead of teeth, no more than round and blackish stumps.

"After the breaking of the teeth, the gums, as may be readily conceived, continue to be diseased. Around the stumps, they present a slightly flabby and sanious condition, which renders the breath, not horribly mephitic, as in the case of scorbutic, mercurial, and certain other ulcerations, but insipid, sickening, sometimes to such a degree that the atmosphere of the cutting shops presents a repulsive odor, and almost occasions nausea.

"This inflammation produces neither heat, hemorrhage, nor itching, and no pain to the touch and during mastication; besides, the majority of the cutters, although they have had it for long years, are ignorant of their affection."—*Presse Medicale Belge.*

---

(Translated for the Dental Register from "L'Art Dentaire.")

### DENTAL FISTULAS.

*Temporal Fistula, occasioned by one or more molar teeth abnormally developed near the ear. Operation. Cure.*

I operated in this manner: The fistula being on the left, the animal was necessarily laid upon the right side; a bundle of straw placed under the neck and shoulders raised the top of the head; the hair having been cut off, I made in the temple a crucial incision of from three to four centimetres, and detached, with the double-sage leaf,* the skin of the osseous wall, to which it firmly adhered. The skull being rendered thin in this place by the presence of the foreign body, I easily removed a piece of it with a strong pair of forceps and the frog.† I afterward endeavored to introduce into the fistula thus enlarged, solid levers of different forms and sizes, and succeeded, without too great difficulty, in inserting one strong enough to make a few twisting movements, so as to *unbezel*‡ the tooth which I had to extract. Then shortening as much as possible the arm of the lever between the point of support and the resistance, in order to operate upward and keep from compressing the brain, I succeeded the first time in fetching out a sufficient portion of the foreign substance to be seized with the pincers. But the tooth could not yet be extracted with this instrument, and I had to introduce the lever anew, and pass it around in all directions to effect the evulsion of this production. The extraction of this abnormal tooth left a deep cavity in the osseous wall, which was almost entirely smooth. I assured myself that there was no tooth wanting in the mouth.

All the temporal fistulas that I have observed, were occasioned and supported by the presence of one or more molars, which filled the place of foreign bodies; and those which I have extracted all had the crown turned toward the skull; it

* Annals of Veterinary Surgery. † Ibid. ‡ Ibid.

is always this part which is first brought out, and, though usually deformed, it is not difficult to distinguish it from the root. The enamel which envelops it, prevents it from being confounded with the root, which has no enamel. The root takes very diverse forms, but in general it is round, swollen, and distorted, like that which I send you with this note.

Of the fourteen horses upon which I have operated, thirteen have been completely cured at the end of from ten to fifteen days, without any other care than cleanliness. The fourteenth has been operated upon twice, with an interval of three months, and each time I have extracted a molar. Instead of the fistula, he carries at present a soft tumor, a cyst, of the size of a small egg, the inner wall of which secretes a gluey, albuminous, and transparent liquid. When the sac is full, the liquid flows out through a very fine duct which opens toward the middle of the anterior rim of the ear. I have pierced this tumor several times, and injected tincture of iodine into it; I have introduced the hot iron into it; have passed a seton into it, without result; the seton being removed, and the openings cicatrized, the abnormal secretion soon re-appeared. It is to be presumed that a third tooth is tending to make its way through by the same opening.

I might have enlarged more, and considered some particulars relative to each of the cases presented to my observation and the different modes of operating which I adopted; but I think that the details which precede, will suffice to give to my *confreres* who may not yet have examined it, a knowledge of the temporal fistula and of its treatment.

I now inquire why the temporal fistula always has its seat in the same place—why it shows itself on the right or the left side? Why do the teeth, which are the determining causes of the fistula, always take the same direction? Why do they push up toward the top of the head, rather than show themselves in the mouth? I shall not attempt to solve these questions; I confine myself to propounding them. I shall say only as to the direction impressed upon these productions, that it seems to me to be the result of a kind of retroversion of the dental embryo. Accept, etc. J. MACORPS,

Veterinary Surgeon to the Government.

### ARTIFICIAL DENTURES.

BY DR. JOHN ALLEN.

Lower sets of artificial teeth are often more difficult of adaptation than upper ones, owing to the great amount of absorption that may have taken place on the lower jaw, leaving scarcely any ridge or foundation for them to rest upon, and in some instances the place where the ridge ought to be, there is only a deep hollow, with muscles, ducts and glands, almost closing over it on either side. These tend to buoy up the denture and keep it afloat in the mouth, which renders it useless, if the plate is so wide as to rest upon them.

In order to overcome the difficulty when such cases occur, the impression should be taken with great care to prevent the inner folds of the cheek from overlaying the alveolar ridge, or hollow. These folds can be easily raised and moved outward with the fingers when the impression is being taken. After the impression has become hard, it should be trimmed so as to form a very narrow bed, from which to form the plate. When the submaxillary ducts, sublingual glands, or their connecting fibrous tissues, raise above the hard ridge, when the tongue or muscl, s are moved, the base should be so narrow as to allow them to pass up on the inside of the plate and the teeth without dislodging the denture. For such cases the plate should be very thick and narrow, with smooth round edges, and the teeth should be quite thin.

### HOW LONG, AFTER THE EXTRACTION OF NATURAL TEETH, BEFORE AN ARTIFICIAL DENTURE SHOULD BE INSERTED.

From one to two weeks the writer deems sufficient time, under ordinary circumstances, for the patient to go without teeth; and in many instances even a shorter period would suffice.

But as there is a difference of opinion upon this point, we will present the different theories and submit to the reader their claims to supremacy. The objections urged to an immediate insertion of a denture are: that the gums will not become smooth and symmetrical if a plate be fitted to them before the alveolar processes have fully absorbed, but will conform in a greater or less degree to the irregularities of the temporary plate, and prevent a good practical result; consequently they advise their patrons to go without teeth at least one year.

This theory we think, erroneous, because the slight undulating surface of the gums which the plate may have occasioned, presents no valid objection ; for a permanent plate can be fitted to them just as perfectly at the expiration of one year, as if a temporary plate had not been worn, and the wearer will have become so well accustomed to artificial teeth, that the second set can be worn and used at once without difficulty, if properly constructed.

Again, the longer the natural teeth have been out, the harder it is for a person to acquire the use of artificial substitutes, for the lower jaw is thrown forward of its natural position, the lips become compressed, the muscles of the mouth and face contracted, and the tongue habituated to certain movements in munching food, which tend to dislodge the dentures when attempting to use them, and the original expression in many instances is lost.

The advantages of an immediate insertion of artificial teeth after the removal of the old ones are,

1st. The patient will acquire the faculty of using them satisfactorily in much less time than if required to go without any teeth until the healing of the gums and absorption of the alveolar processes have taken place.

2d. The original expression can be much better preserved, as the denture prevents that compression of the lips, and contraction of the muscles about the mouth and face, which the absence of the teeth will cause.

Temporary sets should be as skillfully made as permanent sets. There are many operators, however, who insert cheap and rude temporary sets of teeth, who seem to attach but very little importance as to how they are constructed, for they are only intended to be worn a year or two, which is deemed a sufficient apology for poor work. The price usually paid for temporary teeth will not justify the use of the best materials, nor the highest degree of skill and taste in their construction. This is another reason for cheap work.

Here is an error that ought to be corrected, for in this way the patient has but a miserable substitute for the natural teeth, and is often subjected to extreme mortification at their rude and uncomely appearance and want of utility.

Again, the dentist often looses cast by this kind of work, for a poor cheap specimen is often looked upon by others as a fair sample of the skill of him who made it. Although the inference may be very unjust, yet it tells against him. We think reform in this respect would prove mutually advantageous to the patient and operator.

Instead of this cheap graceless work, let the first set be as good as the second, or as near it as the case will admit, and let the charges be based upon terms of equity. Then the patron will be better served and better satisfied than with a rude fixture, devoid of the essential requisites of an artificial denture; for however perfectly formed and adapted artificial teeth may be, they usually meet with stern resistance when first inserted, (especially full sets,) for the tongue, muscles, glands and ducts, all conspire to eject them from the mouth as unwelcome intruders. They seem not to have forgotten the old offenders that used to create such painful sensations in their neighborhood until they were all exterminated, since which time there has been comparative peace.

But the sterner will can subjugate all these opposing forces and establish harmony, provided the dentures are rightly formed, which is the condition of the compromise. The tongue will then acquire more cautious habits, and avoid those movements which at first would send them whirling out of the mouth, for the tongue is the first to repel invasion, it does not like to be restrained. The contiguous muscles, ducts and glands, also require freedom of action and they must not be infringed, for this is nature's law. But if their natural movements and functions are provided for, then the teeth will be permitted to remain quietly in their proper places and subserve the purposes for which they were designed.

But still, even with the most perfect set of artificial teeth there will be in many instances a stiff and restrained feeling and appearance about the mouth, when first inserted, that time only can remove, especially if the natural teeth have long been out. Patience and perseverance will, however, entirely overcome the difficulties that at first appeared so formidable to a new beginner.

The above article contains ideas and suggestions that should be carefully considered by every dental operator.

The idea has been generally entertained, that nine to twelve months should elapse after the extraction of the natural teeth before introducing substitutes. We have for several years entertained the opinion that this is not the best practice. This article presents some of the advantages of inserting teeth soon after the removal of the natural organs. It is true that when a plate is inserted immediately after the extraction of the teeth, the gums may not absorb as smoothly and evenly as though a plate had not been worn, while the process of absorption was going on. This, however, is a decided advantage and not a disadvantage as has been supposed. A plate will retain its position far more firmly on a rough than a smooth gum, if as well adapted; and it is about as easily adapted.

When a person is without teeth for a considerable time he, of course, loses facility of use. After the teeth are all removed there is no resting point for the jaw, and by a relaxation of the muscles, it attains a lateral motion, that is always exceedingly annoying, when the patient attempts to use the teeth for mastication. We are familiar with cases of this kind, where teeth have been worn for several years, and affording but little service for mastication. We have yet to find the first case in which teeth were inserted soon after the natural organs were removed, that did not answer a valuable purpose for mastication. It is a popular notion that if plates are put upon the gums before they are healed, they will be made very sore, and continue so.

Experience warrants us in saying that no more soreness is experienced by wearing plates than though they were not worn.

When the teeth have been out for a considerable time the tongue attains a latitude of movement that is rather difficult to control, when artificial teeth are placed in the mouth; under such circumstances there is difficulty in speaking. A patient after wearing a temporary set of teeth in the mouth for nine months or a year, which by absorption of the gums fit very imperfectly, will appreciate a good, permanent, well-fitting set of teeth, and will use them with facility at once.

[Ed.

### CASE OF ABSCESS OF PAROTID GLAND.

REPORTED BY J. LYKINS, M. D., OF KANSAS CITY, MO.

In August, 185–, Mr. J. J. a respectable farmer, aged 45, was attacked with congestive fever, ushered in by a chill. Upon the invasion of second chill, I was called to see the patient. Found the extremities cold, pulse hardly perceptible, patient unconscious, passing involuntary stools, etc. By the most prompt and energetic treatment, after three hours effort, pulse rose and fever set up.

2nd day.—Kept patient under the influence of free portions of quinine, remained with him three or four hours, preceding and during the time of expected chill; patient escaped both chill and fever, and was dismissed as requiring no further treatment.

4th day, 4 o'clock P. M.—Was called in great haste to the patient, found him suffering most intense pain, and upon examination found its seat to be the Parotid Gland, which proved to be slightly enlarged and too painful to be touched. The patient having been subjected to a vigorous treatment of sub. mur. hydrg. and sulph. quin. in order to break up the fever, was put upon the use of saline cathartics and the usual lotions externally.

6th day.—Jaws fixed, gland greatly enlarged, general tumefaction much increased, jaws too rigid to admit of anything entering the mouth except fluids; patient delerious. Prescribed castor oil to be followed by quinine, external applications the same.

7th, 8th, and 9th days.—Treatment continued, left eye closed by swelling, articulation suspended.

10th and 11th days.—Prognosis hopelessly unfavorable; called in Drs. Boggs and Caldwell, both of whom agreed without hesitation, in pronouncing the case one of abscess of the parotid gland, supervening upon the congestive stages through which the patient had passed during the first attack.

12th and 13th days.—Tumor purple; first indications of pus deeply seated.

14th day.—Minute portions of purulent matter escaping from the ear, with indications of pus pointing over the centre of the gland.

15th day.—Introduced lancet, pus was reached easily, but escaped slowly.

16th day.—Patient feeble, discharging enormous quantity of dark, morbid bile, from the bowels; no relaxation of the jaws; treatment continued.

17th day.—Plunged lancet into the tumor, anterior to the ramus of the lower jaw, below the masseter muscle; as before reached the pus; but it would not flow. Flax-seed meal poultice applied.

18th, 19th, and 20th days.—Pus tenacious, escaping slowly, skin sloughed away below the ear to the angle of the lower jaw, exposing viscid pus adherent by cellular tissue, and resembling end of grass rope.

21st and 22d days.—Jaws slightly relaxed, swelling abated partially, patient desires food, is more rational.

23d, 24th and 25th days.—Slough enlarged, discharge exceedingly offensive.

26th, 27th and 28th days.—Takes soup freely, and desires meat, improving; tonics continued.

29th, 30th and 31st days.—Opening under the ear enlarging pendant from which hangs the suppurating inferior portion of the parotid gland. Attempted to cut it away with Surgeons' scissors; so resistant as to cause the scissor blades to chew; left side of the face paralysed, and cheek drawn aside.

32nd and 33rd days.—Patient rapidly improving, jaws are partially moveable; succeeded in removing a portion of destroyed gland, which brought away with it the trunk of the portio dura and its branches temporo facial and cervico facial, the external carotid artery, etc.

34th day.—Patient continues to improve; gland slowly coming away. This continued many weeks; at last the ulcer healed, and the patient regained his health.

Happily for us this patient now resides near our city, and to an examination of the cicatrix, and to the evident absence of the parotid gland, I beg leave to invite the attention of the Editors of this Medical Journal. I do this with the more interest as Dr. Titus Deville, in the Chicago *Medical Examiner*, has denied that the parotid gland is liable to disease, and has thereby in my opinion, convicted himself of the ignorance which he has charged upon Prof. Brainard.

Dr. Caldwell, of Independence, a most intelligent physician at the time of the above related case, informed me that he had in this country witnessed two cases of abscess of parotid gland, consequent upon congestive fever, both of which proved fatal.—*Kansas City Med. and Surg. Review.*

### USE OF CHLOROFORM AND ACONITE IN NEURALGIA.

In a letter to the *Medical Times and Gazette*,, Dr. Gueneau de Mussy writes .—

" For more than three years I have prescribed in neuralgia of different regions ; but here I want chiefly to allude to the most common and severe of neuralgic pains—the facial neuralgia—in which I have derived from the above named remedy sometimes a complete and permanent cure, and always an almost immediate and considerable relief. In such cases I apply it directly to the gums of the side affected, where numerous divisions of the fifth nerve are most superficial. When the neuralgia is idiopathic—*i. e.*, unconnected apparently with any other disease—the formula is :—Two parts of spirit of wine or eau-de-colonge, one of chloroform, and one of tincture of aconite. The finger, covered with a piece of lint or soft thick linen, is dipped in the mixture and rubbed gently against the gum for a few minutes. I do not use a sponge, because it would take up too much of the liquid, which, by pressure, might drop into the mouth.

" When the pain is connected with some organic disease, as a deranged tooth, chronic inflammation of the gums or of the sockets, or, as I have observed, superficial necrosis of the bone, I have found the tincture of iodine a very beneficial substitute for spirit of wine in the formula above.

" The infra-orbitary branch being the most commonly affected, it is chiefly in neuralgia in this part the application has been successful ; but by no means exclusively so—it answers very well in pain of the lower branch ; and I have observed some very severe cases of supra-orbitary neuralgia, in which the same application has been attended with an equally satisfactory result.

" This shows, moreover, that the sedative agent may produce its effect, as the irritating one so frequently does, on a part distant from the spot on which it was applied in the same, and even in a different branch of nerve."—*Chicago Medical Journal.*

### PERISCOPE—FATALITY FROM CHLOROFORM.

Dr. Kidd, who has given much attention to the subject of chloroform, has observed that deaths attributable to its inhalation have occurred more frequently during the performance of the minor surgical operations. The statistics of deaths from chloroform certainly show a much greater proportion in the performance of trifling operations, as of 85 fatal cases in which the nature of the operations was recorded, 10 were extractions of teeth, 14 removals of toe nails and operations on phalanges, while of this number none occurred in the performance of the large amputations, resection or ligature of large arteries, etc.

Dr. Kidd has therefore hastily concluded from these results, that "chloroform is safer in large than in small operations." He seems to have overlooked the fact of the vastly greater frequency of the performance of small operations, and of course the more frequent administration of the anæsthetic, which is, we believe, sufficient to account for the apparent greater fatality attending minor operations.

Dr. Kidd estimates the number of deaths from inhalation of chloroform to be about one hundred. We think that if this number had been quadrupled it would more nearly approximate the truth. Chloroform never gained general confidence in this country, and its use has within the past few years rapidly declined, yet the deaths referable to it would probably equal one half of Dr. Kidd's entire estimate of fatality from it.

The European origin of chloroform inhalation and its distinguished authorship, has given it a confidence which cannot long be maintained in the face of such uncontrollable mortality, and while the causes of sudden death from it are so little understood.—*Medical and Surgical Reporter.*

---

*Alarming Symptoms caused by the Displacement of Artificial Teeth during Etherization.*—Dr. Warren reported the following case: He lately had occasion to etherize a lady, 35 years old, in order to examine a painful tumor of the leg. She came quietly under the effects of the ether, but did not arouse afterward. The pulse was good, and there were no symptoms of dyspnœa. She gradually became purple in the face, was quite insensible, and seemed to be passing into a dying state. Introducing his fingers into the mouth, in order to draw the tongue forward, Dr. W. found a complete set of upper teeth, attached to a gold plate, deep in the fauces. This was removed, the fauces irritated, the patient rubbed, &c., and at last vomiting was brought on, and she revived. She soon became violently delerious, uttering shrill cries, and beating herself for an hour and a half. For the next two hours she was in a croupy state, from the violence of her efforts, but in the course of the evening she gradually recovered, though she remained hoarse for two days. Dr. W. observed that the accident was one likely to occur under such circumstances, and showed the expediency of removing artificial teeth before proceeding to etherize a patient.

Dr. Parks alluded to a case which he had already reported to the Society, in which a patient experienced severe symptoms of suffocation, caused by unconsciously swallowing a set of false teeth, during sleep, which had lodged behind the glottis. The symptoms were immediately relieved by the removal of the foreign body.—*Boston Med. & Surg. Journal.*

### ADVENTURE WITH THE FILE.

BY GEO. S. FOUKE.

Experimental dental life is *sacred.* But there are occurrences of a character that may be spoken of without violating professional obligations of this *sacredness* to our actual or *unactual* patients. *Unactual patients* are such as call on the dentist to have their teeth *fixed* according to their *own ideas,* and not in accordance with the *notions* of the *tooth doctor.* This class of patients, however, are, thanks to the spread of "*true* dental knowledge," becoming "small by degrees and beautifully less."

There are some things, then, we *dare* speak of, that, if brought out in stereoscopic life-like realities, would interest, if they did not instruct, the professional as well as the unprofessional eye. We often recur, in our imagination, to an adventure we had once with the file; and inasmuch as this instrument is still a *vexata questio* among dentists, we shall ask the Examiner to look at a *stereoscopic view* of this bit of experience, in a by no means uneventful dental life, hoping its bearing on the disputed question will be fairly appreciated and duly improved.

In June, 1855, a mother and her daughter (smartly past *sweet sixteen*) came to us for dental service for the latter. They were very genteel in appearance, and commanded our respectful attention. Indeed their calm dignity and composure of deportment had the effect to awe our natural *taciturnity* into involuntary *silence*. The mother announced very soon the object of their visit. "My daughter wants her teeth *plugged*." *Pantomimically* we conducted the fair one to the chair. She maintained the utmost self-possession, and we thought here was one ready to pass *unnerved* the dental ordeal about to be enacted. The mother took a position to view serenely the course of "coming events." *These* (fortunately!) did not "cast their shadows before!" If they *had*, this *picture* would never have been.

The glass mirrored what even a *true* dentist does not like to see—an entire denture almost ruinously decayed for the want of timely operations, which would have saved the teeth for the comfort and well-being of their possessor.

As we were gazing, *sickened* at the sight, we imagined we heard *Miss Pulpa Dens* faintly sighing, and saying: "Ah, thou friend of the *Dens family*, thou seest the ruin that has come upon my brothers, *Enamel* and *Dentine Dens*, in consequence of the long and cruel neglect of those whom nature has assigned as our possessors and guardians! Pity us; save us, if you can, from utter destruction! We belong to those who know not our real wants. They have strange ideas about the teeth and dentists. They think dentists ought to have to earn their money 'like other people!' They think *cavities* should be large, then the dentist would not only have a *good*, but a *safe* place to deposit his *surplus* gold! O doctor, pity us, and do something to save us, if you can!"

As we removed the mirror from the mouth, the mother again spoke: "Doctor," says she, "it is the front teeth that need *plugging*—not the back teeth, doctor; they are not decayed enough yet for *plugging;* it is the front teeth that want *plugging!*"

Shades of Hayden! Not decayed enough yet! We felt some relief, however, in having our attention limited to the front teeth; and although these were decayed *infradentinally* on all proximates, yet we hoped, as they possessed great *masculinity* of form and structure, we might be able to benefit them by plugging; so we braced ourselves up for doing our best in *depositing* some surplus gold.

Our examination showed a craggy piece of epienamel, overhanging the proximate of its incisorial neighbor, and which offered a serious obstruction to operations upon that particular locality. We determined to remove this for the first thing, and picked up a file for the purpose. Turning to our fair patient, we reached out our hand which held the file, at which moment she seized our hand, and uttered a stern command to "stop!" This was quickly followed by "*what is that?*" and "*what, do you plug teeth with that?*" Drawing back from our *patient*, we were just breathing out a faint affirmative, when the mother spoke for the third time:

"Her father, sir, gave us express orders not to allow her teeth to be filed, or to be touched with the instrument. He is very much opposed to filing teeth. Dr. Anti-Fil(e)libuster, a distinguished dentist in B——e, won't file teeth, and thinks it is '*a sin*' to file teeth. He told her father never to allow any dentist to file any of our teeth. If you, sir, *plug* teeth with *that*, (looking *daggers* at the file in our hands,) we had best have nothing done now."

"Certainly, madam, *nothing* had best be done now," responded we, very happy to be thus *foiled* in carrying out our *fil(e)libustering* propensities upon these four wretchedly decayed incisors.

*Wonder* who they found to *plug* those teeth, and to *confirm* them in their prejudice against the *horrid* file?—*Southern Dental Examiner.*

### ADVICE TO PATIENTS.

BY J. S. LATIMER.

Those theorizings on the predisposing causes of diseases incident to the dental organs, are well enough among the *profession;* but they are very far from being of any practical value to our patients. I have reference, here, to those primary causes, such as the intermarriage of persons with jaws and teeth differing greatly in size, by which irregularity is often produced in the offspring, as well as to those conditions of the mother, during gestation and lactation, by which is regulated the amount of earthy salts in the teeth of the offspring.

For the next hundred years, at least, the influence of dentists, in this direction, will be confined to their own households. But, if we can not prevent the *predisposition* to disease, we can recommend to our patients such hygienic treatment as shall (if strictly followed,) be greatly beneficial.

It is not less our duty to our patient than to ourselves, to administer proper advice, even unasked. Patients are seldom properly impressed with the importance of preserving the dental organism perfect, and they are generally grossly ignorant of the causes which produced disease. As a general rule, the attention paid by any set of persons to their teeth, is a fair index of their enlightenment.

In general medicine, taciturnity and secrecy *may* add to the practice of the physician—in dentistry they never can.

If a patient calls upon us with teeth encrusted with tartar, he should be informed how tartar is deposited, and how it produces disease and absorption of the gum and alveolus, by which the teeth are ultimately loosened and lost. He should then be informed of the method of curing and of preventing a recurrence of the difficulty.

No *really professional* man will *urge* his services upon the patient; but, having stated the facts fully and plainly, will await the decision.

If carious teeth are to be treated, and the organs are found to be of a "chalky" texture, or, if any are so badly decayed as to render the chances of preservation, for any considerable time, rather dubious, the patient should be informed of the facts, in order that the operator and his profession shall not be held responsible for more than is just.

"*Cleanliness akin to godliness,*" is the motto which should be urged upon our patients; for, without it, there is no salvation for our plugs, however perfect they may be; and, when our plugs fail, from whatever cause, the teeth and our caste are lost together.

But, right here is a difficulty; the profession is not consistent with itself; in other words, operators differ in opinion with regard to the best method of preventing decay, etc. One recommends hard and another soft brushes, while a third ignores brushes altogether. And the same difference of opinion prevails with regard to the employment of dentrifices, the removal of temporary teeth, the treatment of exposed dental pulps, etc.

The tendency of this disagreement is to lessen the confidence of the public in dentists and dentistry.

I am aware that dental schools have a tendency to enlighten dentists and to produce uniformity of opinion, and that the periodicals of our profession serve to break down those theories which are not founded in science ; yet, in order that the discussion of the theories on which our every-day advice is founded, I propose to give the principles on which *my* advice is based, hoping others will follow me.—*Southern Dental Examiner.*

### PRACTICAL HINTS.

BY J. D. WHITE.

Almost daily experience shows that there is very little sound judgment exercised in the operation of extracting teeth, especially in cases that apply for the operation in the alleviation of toothache. When a patient applies for the extraction of a tooth, while suffering from pain, and we can relieve the case without removing the tooth, as a *rule* we do so. There are very many circumstances to be taken into consideration, however. If the patient has never had a tooth extracted and is very young, whether it be a deciduous tooth or a first molar of the permanent set, it is not judicious to attempt its extraction at once, unless it be to prevent some great impending danger. The *moral* influence upon the patient may be such as to injure him much more than the pain from the tooth. It is not unfrequent that the patient has been assured by those in whom he placed confidence, that he would suffer no pain: under such circumstances, the tooth should not be extracted. Palliate the pain, or at least make an effort to do so ; as a general thing, this can be done, if it be from exposed pulp or incipient abscess. If the patient discovers that this can not be done, he will much more willingly submit to the operation without it having any unfavorable influence on the mind. Besides, if the tooth can not be removed with certainty, it should not be attempted, either with young or old ; the dentist always receives much more censure for failing in this operation than any other. Sometimes the patient's nervous system is not in a condition to undergo the operation, especially if it were to be prolonged.

We have not been caught for many years in making more than a second attempt to extract a tooth, owing to the fact that we observe the greatest circumspection. We estimate the chances of getting hold of the tooth or root, and the *nerve* of the patient to endure pain. Before we believed that it was *not* our duty to attempt to extract a tooth for a patient under any circumstances if the patient suffered pain or inconvenience from bad taste in the mouth, or a difficulty in keeping the mouth clean, we got into many a scrape. It would be endless to enumerate the cases we know and hear of, where the dentist has tortured the patient for hours, and been at last obliged to abandon the operation. We make it a practice to wait until the tooth is in a *condition* to be extracted. This may sound like timidity or shrinking from our duty by "tooth pullers," but we have the vanity to believe that we do not often fail to do our duty. It is true that many do not do their duty by attempting what must end in failure. It may be inquired, what does it mean, to get a tooth in a condition for extracting? We will answer, that if a tooth has decayed much below the border of the alveolus, except the merest shell, and the gum and socket are too hard and too strong to be cut away, it is better to break down the projecting shell or crown and wait for the roots to loosen and rise from the sockets, as they will do in time by the morbid condition of the periosteum, and the absorption of the gums and alveolar border. It often happens that teeth are decayed to the pulp before they are fully erupted, and it is difficult to get to the neck with the forceps.

We do not mean that the dentist shall take no risk in attempting to extract a tooth in extreme cases. We always estimate the difference as well as we can between the chances of success in extracting a tooth, and the amount of suffering it may cause by letting it alone. A case or two may illustrate what we mean. A gentleman called to get a tooth extracted a few years since; it was the left inferior wisdom tooth; it was strongly imbedded in a strong and heavy jaw, with little room between the second molar and the ramus of the jaw. It was a case of exposed pulp; we applied the arsenical paste to destroy it, but the patient left us and immediately called upon another dentist to get the tooth extracted, not being satisfied with our treatment. The dentist was a young man, and attempted to extract it. After trying for a long time, he failed to succeed. He called his father, who was a much stronger man, and he failed also, but not until the patient became exhausted, when it was abandoned. The patient was laid up from the effects for several days; we have always operated for the gentleman and his family since. Another case occurred of a lady, who annoyed us very much to get rid of a lower inferior first molar, which was decayed very much, and the gum and alveolus very strong, and the patient nervous, and not able to bear much pain. She finally applied to a dentist, took ether; but the dentist failed to extract it. The parts were much injured in the attempt. The patient applied to us in great suffering; we touched the margin of the gums with nitrate of silver in the stick, which reduced the irritation of the gums and relieved the pain in a few days. We applied the same treatment again, until a considerable portion of the gum was destroyed and absorbed. In this way the sensibility of the parts was reduced, when we cut away the alveolus and extracted the root with ease.—*Dental Cosmos.*

Care of the Sick.—Not to allow a patient to be waked, intentionally or accidentally, is the *sine qua non* of all good nursing. If he is roused out of his first sleep, he is almost certain to have no more sleep. It is a curious, but quite intelligible fact, that if a patient is waked, after a few hours' instead of a few minutes' sleep, he is much more likely to sleep again. Because pain, like irritability of brain, perpetuates and intensifies itself. If you have gained a respite of either in sleep, you have gained more than the mere respite. Both the probability of recurrence and of the same intensity will be diminished; whereas both will be terribly increased by want of sleep. This is the reason why a patient, waked in the early part of his sleep, loses not only his sleep, but his early power to sleep. A healthy person who allows himself to sleep during the day, will lose his sleep at night; but it is exactly the reverse with the sick generally; the better will they be able to sleep.—*Miss Nightingale.*

